# Laparoscopic transabdominal preperitoneal repair of strangulated femoral hernia: Superiority of an unusual emergency surgical approach due to a case

**DOI:** 10.1016/j.amsu.2018.10.014

**Published:** 2018-10-19

**Authors:** Mehmet Tolga Kafadar, Mehmet Ali Gök

**Affiliations:** a Health Sciences University Mehmet Akif İnan Training and Research Hospital, Clinic of General Surgery, Şanlıurfa, Turkey; b Health Sciences University Derince Training and Research Hospital, Clinic of General Surgery, Kocaeli, Turkey

**Keywords:** Femoral hernia, Strangulation, Laparoscopic surgery

## Abstract

**Introduction:**

Inguinal hernia repair is one of the most common operations of general surgery. In order to avoid complicated and urgent cases, performing such operations electively is generally accepted. Otherwise, unforeseen emergency surgical situations accompanied by incarceration and strangulation may occur.

**Case presentation:**

In this article, we present a 45-year-old female patient with strangulated femoral hernia repair that we performed using the laparoscopic transabdominal preperitoneal method, unlike other conventional methods.

**Discussion:**

Early diagnosis and elective surgical treatment have an important role in hernia surgery, especially due to increased morbidity and mortality. Laparoscopic inguinal hernia repair has developed in the recent years as a prominent method and nowadays it is performed much easier than the open method even in urgent and challenging cases.

**Conclusion:**

The transabdominal preperitoneal method has superiority over the conventional method in terms of ensuring that intestinal loop is visible during the strangulated femoral hernia repair and that the feeding of the intestine is intact.

## Highlights

• The TAPP method has superiority over the conventional method in terms of ensuring that intestinal loop is visible during the strangulated femoral hernia repair.
• This method allows us also the possibility of diagnostic laparoscopy for the emergency cases.
• Direct visualization of the incarcerated or strangulated organ and, if necessary, ability to perform resection laparoscopically are significant advantages of this technique.

## 1 Introduction

Femoral hernias originate from the femoral canal below the inguinal ligament, and constitute about 5% of all inguinal hernias. It is 3–4 times more common in women. It can be confused with expanded and painful lymph nodes in the region. Femoral hernias occur through the narrow ring called femoral ring near the major arteries and veins of the legs. The risk of incarceration and strangulation of femoral hernias is high. Thus, intestinal resection is performed more frequently than other inguinal hernias [1]. Repair is more difficult than inguinal hernia because of deeper cavity where hernia occurs and proximity to the major vessels of legs, and the risk of reoccurrence is higher. Diagnosis of incarceration or strangulation can be finalized only during surgical exploration because there may be no significant association between intestinal viability and clinical manifestations. Morbidity and mortality are associated with the feeding of incarcerated bowel, and resection has a significant effect on morbidity [2]. Herein, we present a strangulated femoral hernia case which was treated by laparoscopic method in emergency conditions without resection.

### 1.1 Presentation of case

**Fig. 1:**
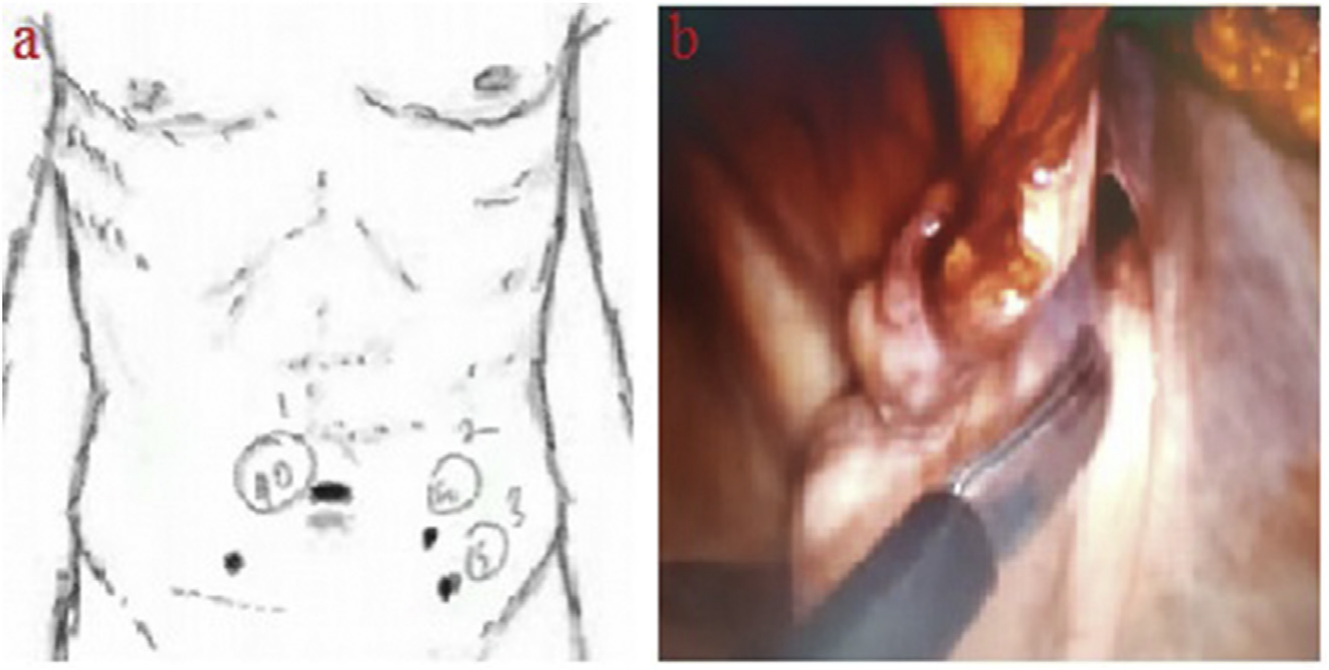
Laparoscopic transabdominal preperitoneal femoral hernia repair; positioning of the trocars (a), appearance of the hernia sac (b).

**Fig. 2:**
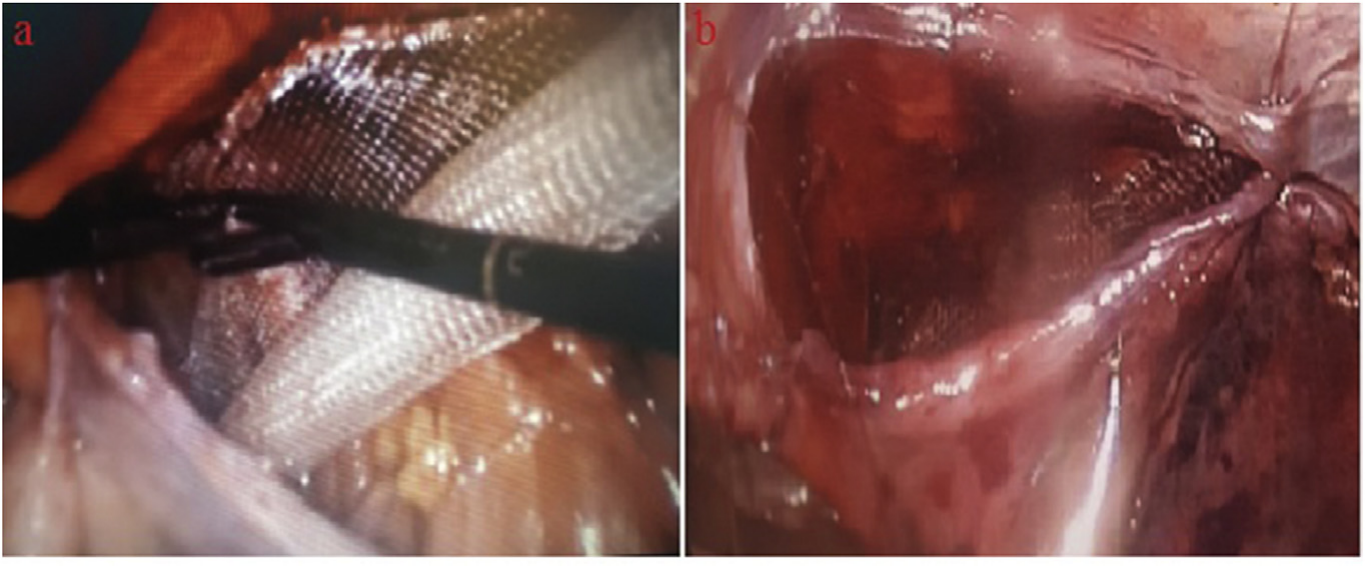
Placement of the mesh (a), repair with the stratafix (b).

A 45-year-old female patient was admitted to the emergency department with complaints of swelling and pain of the right thigh. The patient expressed in the anamnesis that she has an inguinal swelling for about 1 year, the swelling collapses when applying pressure on it by hand, however, that it did not disappear and pain increased within the last 24 hours. She did not express any previous operation in the anamnesis. During physical examination, a rigid painful swelling of approximately 4 cm was palpating in inferior of the right inguinal ligament. An edematous aperistaltic bowel segment of the right inguinal canal has been reported based on an ultrasonographic imaging and an urgent operation was decided. Under general anesthesia, the abdomen was entered by the laparoscopic method via umbilical 1 × 10-mm trocar and left abdominal lower quadrant 2 × 5 mm trocars (Fig. 1a). During exploration, a distal jejunum loop of approximately 10 cm has entered into the hernia sac through the right femoral canal. The strangulated bowel loop was reduced into the abdomen in a controlled manner with the help of a bowel clamp and external manual manipulation. We reached to the conclusion that circulation through the strangulated bowel loop has improved after a while and performed no resection. The peritoneum was marked over the hernia defect (Fig. 1b) and it was cut using a scissor in the shape of a bow. The peritoneum was dissected. A prolene mesh was placed in the preperitoneal area where the defect is localized and it was not fixated (Fig. 2a). The peritoneum was closed using the non-slipping suture STRATAFIX ™ (Knotless Tissue Control Device- Ethicon, Johnson and Johnson, Somerville, New Jersey) (Fig. 2b). Stratafix sutures provide more strength and security, more consistency and more efficiency than traditional suturing. The patient had no problems during the postoperative period and was discharged in healthy status on postoperative day 3 following stool discharge and to tolerate oral intake. No recurrence of hernia was seen during the follow-up period of 12 months. Informed consent was obtained from the patient who participated in this case.

## 2 Discussion

When potential complications of incarceration and strangulation are compared with minimal risks of hernia surgery, early repair of the inguinal hernias is clearly required. Because of these reasons, early diagnosis and elective surgical treatment have an important role in hernia surgery, especially due to increased morbidity and mortality in elderly patients. Small bowel obstructions, incarcerations and associated bowel resections significantly increase morbidity. In addition, prolonged hernia and irreducibility time, delayed admission to the hospital and the presence of comorbidities lead to increased postoperative complications and mortality rates. Admission to the hospital in late stage is particularly affecting the decision for resection [3]. While necrotic bowel resection rate was 7% in patients admitted to the hospital within the first 24 hours after complaints developed, it was found 33% for those admitted after 48 hours. This delay may occur because of neglect of the patient, or the clinician may have made a diagnostic error (12–33%) [4].

Although the presence of hernia is generally accepted as an adequate indication for elective repair, strangulated inguinal hernias are still located at the top of the reasons, which constitute an acute abdomen picture and are the most common surgical interventions. It is accepted that femoral hernias constitute a risky group within the inguinal hernias and often cause complicated admissions. The developmental mechanism of the femoral hernias is not clearly understood yet. Because of increased intraabdominal pressure, the pelvic peritoneum is dragged along with the preperitoneal adipose tissue when it passes through the femoral ring. The hernia sac travels down the femoral veins in front of the thigh. In females, femoral hernias may also develop due to weakening of the pelvic floor muscles secondary to previous deliveries. Such patients often complain of inguinal swelling, pain and feeling of contraction. Although most of them are diagnosed during physical examination, ultrasonographic and tomographic imaging help to diagnose hernia of patients who have symptoms but not physical examination manifestations. Ultrasound imaging also allows the incarcerated hernias to be separated from a pathological lymph node, a palpable rigid mass or lipomas [5].

Twenty to 40% of the patients with femoral hernias apply to emergency departments with a picture of incarceration and strangulation. The majority of patients are over 60 years of age and, compared to males, more femoral hernias are seen in the right femoral region of females than in the left [6]. Studies indicate that the most common incarcerated inguinal hernias are femoral hernias (approximately 69%). Narrow femoral canal is indicated as the reason of this [7].

Although many methods have been described in the literature to repair inguinal hernia, there is no any accepted standard method yet. Open or laparoscopic method can be used for treatment. Patched repair performed using the open method can cover the areas where the direct and indirect hernias come out, but might not cover the hole of femoral hernia. On the other hand, a laparoscopic repair may cover all three (direct, indirect, femoral) hernias internally. Since femoral hernias are seen mostly in women, it is safer to repair the inguinal hernias in women by laparoscopic method. When a laparoscopic repair performed using the right technique, this will not only repair the existing hernia but also prevent the other two types of inguinal hernia that might occur in the same side in the future [8].

There are two methods used commonly for laparoscopic inguinal hernia repair. One of them is the transabdominal preperitoneal (TAPP) hernia repair and the other is the total extraperitoneal preperitoneal (TEP) hernia repair. Although the laparoscopic approach for elective inguinal hernia repair has been accepted in the surgical practice and becoming a more and more commonly used method day by day, the use of this technique for incarcerated or strangulated inguinal hernia repair is still controversial. The technical difficulties experienced during reduction of the hernia sac and its content to the abdomen and the increased risk of iatrogenic organ injury are the most important reasons limiting the laparoscopic approach. On the other hand, direct visualization of the incarcerated or strangulated organ and, if necessary, ability to perform resection laparoscopically are significant advantages of the technique [9]. We have performed the TAPP method for our case, have not performed an intestinal resection and have not experienced any technical problem. In our case, there was no need to perform laparoscopic resection, if required, laparoscopic resection could be performed in the same session. Before and after reduction, we were able to observe the viability of jejunal segment with laparoscopy.

It has been indicated that similar results are obtained when compared the laparoscopic hernia repairs to open surgery. In addition, it has advantages such as lower risk of postoperative pain and infection, ability for patients to return to daily life earlier and a better cosmetic appearance. During emergency hernia operations, more complications may occur compared to elective cases. Minor complications include wound infections and hematoma, ecchymosis, urinary retention, recurrences, hydrocele, nerve cuts, and nerve compression. Major complications that might occur include hemorrhage, vas deferens cuts in men, testicular atrophy, intestinal or bladder injuries [10]. In our case, no postoperative complication was observed.

## 3 Conclusion

Strangulation can only be diagnosed finally following a surgical exploration. The TAPP method has superiority over the conventional method in terms of ensuring that intestinal loop is visible during the strangulated femoral hernia repair and that the feeding of the intestine is intact. This method allows us also the possibility of diagnostic laparoscopy for the emergency cases. When there is a need experienced surgeons can easily perform laparoscopic resection and anastomosis in patients with intestinal necrosis or perforation.

## Informed consent

Written informed consent was obtained from the patient for publication of this case report and accompanying images. A copy of the written consent is available for review by the Editor-in-Chief of this journal on request.

## Provenance and peer review

Not commissioned, externally peer reviewed.

## Ethical approval

The study is exempt from ethnical approval in our institution.

## Sources of funding

The authors declared that this study has received no financial support.

## Author contribution

Concept - MTK; Design -MTK.; Supervision – MTK, MAG; Resource - MTK.; Materials –MTK, MAG; Data Collection and/or Processing – MTK, MAG.; Analysis and/or Interpretation -MTK.; Literature Search - MTK.; Writing Manuscript - MTK.; Critical Reviews – MTK.

## Conflicts of interest

No conflict of interest was declared by the authors.

## Research registration number

This study is not a human study.

## Guarantor

Mehmet Tolga Kafadar.
